# Concurrence of CareStart™ Malaria HRP2 RDT with microscopy in population screening for *Plasmodium falciparum* infection in the Mount Cameroon area: predictors for RDT positivity

**DOI:** 10.1186/s41182-019-0145-x

**Published:** 2019-03-01

**Authors:** Rene Ning Teh, Irene Ule Ngole Sumbele, Gillian Asoba Nkeudem, Derick Ndelle Meduke, Samuel Takang Ojong, Helen Kuokuo Kimbi

**Affiliations:** 1 Department of Zoology and Animal Physiology, University of Buea, Buea, Cameroon; 2 Department of Social Economy and Family Management, Higher Technical Teachers’ Training College, University of Buea, P.O. Box 249, Kumba, Cameroon; 3 Clinical Diagnostic Laboratory, University of Buea, Buea, Cameroon; 4 Department of Medical Laboratory Sciences, The University of Bamenda, Bambili, Cameroon

**Keywords:** *Plasmodium falciparum*, Malaria, Children, Microscopy, CareStart™ Malaria HRP2 pf rapid diagnostic test, Anaemia, Cameroon

## Abstract

**Background:**

Malaria remains a diagnostic challenge in many endemic communities. Although rapid diagnostic tests (RDTs) are presently widely used for malaria diagnosis, there is a dearth of information on post-marketing surveillance on its efficacy in Cameroon. The present study evaluated the performance characteristics of CareStart™ Malaria HRP2 (histidine-rich protein 2) antigen (Ag) RDT in diagnosing *Plasmodium falciparum* infection in the Mount Cameroon area and predictors associated with RDT positivity.

**Methods:**

The CareStart™ Malaria HRP2 *Plasmodium falciparum* (G0141) Ag RDT was evaluated in a cross-sectional community-based survey involving 491 children of both sexes aged 6 months to 14 years between April and May 2018. Malaria parasitaemia was confirmed by light microscopy. Sensitivity (Se), specificity (Sp), positive (PPV) and negative (NPV) predictive values of the RDT, and the corresponding accuracy and Kappa value (*κ*) were determined using microscopy as the gold standard. Haemoglobin (Hb) concentration was obtained using an auto-haematology analyser. Results were compared using the chi-square test and associations between predictor variables, and RDT results were assessed using logistic regression analysis.

**Results:**

Microscopically confirmed malaria parasite prevalence was 27.7%, and geometric mean density was 187 parasites/μL of blood (range 70–1162). Se, Sp, PPV, NPV and accuracy were 82.4, 76.6, 57.4, 91.9 and 78.2%, respectively. Sensitivity depended on parasitaemia and reached 96.1% at densities ≥ 200 parasites/μL of blood. The accuracy of malaria parasitaemia (as assessed by the area under the receiver operating characteristic curve) to predict malaria by RDT was 75.4% (95% CI 70.6–80.1). The agreement between microscopy and RDT was moderate (*κ* = 0.52). RDT positivity was significantly associated with fever (*P* < 0.001), children less than 5 years (*P* = 0.02), history of fever within a month (*P* < 0.001) and anaemia (*P* = 0.002).

**Conclusion:**

The overall concurrence of CareStart™ Malaria HRP2 pf Ag RDT with microscopy in the detection of *P. falciparum* infection is moderate and is most useful at parasitaemia ≥ 200 parasites/μL of blood and presentation with fever. While RDT is effective as a diagnostic test for confirmation of clinical cases of malaria, its applications in population screening with a higher proportion of asymptomatic cases are limited.

## Background

In malaria-endemic countries, many *Plasmodium falciparum* infections are asymptomatic. Falciparum malaria continues to negatively impact human life and fragile economies. Globally, malaria is still a public health concern as 445,000 deaths were caused by malaria in 2016 and Cameroon alone accounted for 3% of this number [1], despite the control measures put in place [2–5]. The current control strategies adopted by the National Malaria Control Program (NMCP) in Cameroon include the intermittent preventive treatment in pregnant women, free treatment of uncomplicated malaria in children under five with artemisinin-based combination therapies (ACTs), indoor residual spraying and more recently free distribution of long-lasting insecticidal nets [1]. Despite these control measures put in place in Cameroon, malaria remains the major cause of morbidity and mortality, with over 90% of the population at risk of the disease [6], accounting for about 48% of all hospital admissions and 30% of all hospital deaths [7]. However, the World Health Organization (WHO) [8] cites Cameroon among the moderate to high burden 21 countries with overall case numbers exceeding 300,000 indigenous cases in 2017. In 2014, WHO [9] cites Cameroon as one of the African countries with insufficient consistent data to evaluate malaria trends and this indicates the likelihood of an underestimate of malaria prevalence. One of the reasons for this insufficient data is probably linked to undiagnosed and untreated cases of malaria as the disease remains a diagnostic challenge to laboratories in endemic countries including Cameroon [10, 11]. Malaria can be diagnosed in several ways which include presumptive diagnosis using the signs and symptoms associated with it, demonstration of the parasite, its parts or soluble products in body fluids such as *Plasmodium falciparum* histidine-rich protein 2 (PfHRP2), that can be captured by monoclonal antibodies raised against these antigens in the form of a RDT [12].

Some communities in malaria-endemic areas lack healthcare facilities, and diagnosis of malaria relies predominantly on its clinical presentation which is nonspecific. Although presumptive diagnosis of malaria is less expensive [13], the accompanying prescription could lead to the treatment of patients without malaria [14], over prescription of antimalarial drugs [15], thus contributing to antimalarial drug resistance [16]. It is worth noting that WHO recommends parasite-based diagnosis first for older children, adults and all suspected cases of malaria regardless of patient age [17].

The examination of Giemsa-stained blood smears for the detection of malaria parasites using light microscopy therefore remains the gold standard for malaria diagnosis, as it provides information on both parasite species and density [12]. However, microscopy requires basic laboratory infrastructure, quality equipment and reagents and is labour intensive and needs a trained technician [10, 18]. In order to overcome the deficiencies of light microscopy, RDTs have thus been designed as alternatives. RDTs require little training, produce rapid as well as prompt results after 15 to 30 min [19], require no laboratory infrastructure and therefore allow them to be used in the most remote settings. The use of malaria RDT has expanded both in endemic and non-endemic settings, with over 60 different RDT brands and more than 200 developed products [17]. Yet, results from field trials suggested highly variable field performance. It is worth noting that newer diagnostic techniques such as amplification of parasite DNA with polymerase chain reaction are specific and can detect low concentrations of parasites but take time and require specialised equipment and are thus not suitable in most field settings.

In 2008, WHO and the Foundation for Innovative Diagnostics (FIND) jointly implemented an evaluation programme, which coupled product testing with a post-purchase lot verification service, to assess, review and compare the performance of malaria RDTs in a standardised manner [19]. Until recently, the WHO-FIND Lot Testing Programme included only limited assessment of RDT buffer and accessories [20]. Even with WHO’s product testing programme, gaps still exist in quality control and quality assurance. With respect to product testing, manufacturers can produce (or procure) RDT batches exclusively for submission to the product testing programme [19]. Moreover, procuring excellent quality RDTs does not necessarily guarantee good field performance because factors such as shipping, handling and storage could affect the RDT accuracy [17].

In many malaria-endemic countries, post-marketing surveillance is non-existent and there are no practical tools that can be used by central reference laboratories or at point-of-care to find out if RDTs are still performing optimally after delivery. Despite persistent high malaria prevalence, and the continuous influx of RDTs into our markets, the surveillance of RDTs in the Mount Cameroon area seems virtually ignored, hence making this study necessary.

The CareStart™ Malaria HRP2 (G0141) Ag RDT is inexpensive, stable at high temperatures (40 °C) and mostly used in health units in the Mount Cameroon area [21]. It contains a membrane strip pre-coated with mouse monoclonal antibodies specific to histidine-rich protein 2 (HRP2) antigen of *P. falciparum*. The antibodies are mixed with colloid gold which conjugate and react with the HRP2 antigen in the patients’ samples. The HRP2 is highly abundant and heat stable; however, the HRP2 antigen remains in circulation for up to 4 weeks after the malaria parasites have been cleared [22]. Although, the CareStart™ Malaria HRP2 pf Ag RDT is being used in the Mount Cameroon area in diagnosing malaria, there is little knowledge on its up-to-date performance characteristics.

So far, several studies conducted in various parts of the country have determined the sensitivity and specificity of assorted brands of malaria RDT kits, including the CareStart™ [21, 23, 24], but there is no up-to-date reporting on the concurrence of RDT with microscopy in field applications in non-clinical settings in the Mount Cameroon area. Therefore, the objective of this study was to assess the performance characteristics of the CareStart™ Malaria Pf (HRP2) Ag RDT (G0141) in population screening in a community setting as well as identify the predictors of RDT positivity in this malaria-endemic area.

## Methods

### Study sites and population

The study was carried out in Dibanda, a rural community located in the Mt. Cameroon area, Fako Division of the South West Region of Cameroon between April 2018 and May 2018. The coordinates of Dibanda ranged from altitude 358 m, latitude 04°06.447′ N, longitude 009°18.725′ E to 400 m, 04°07.179′ N and 009°18.464′ E. The study community comprised of seven residential quarters spanning the entire Dibanda community. The community has no Health Centre and the closest centre offering health services, found in the next town Mutengene, is the only government-owned institution that offers affordable health services to both the Mutengene community and other neighbouring communities. This study area is subjected to a Cameroonian-type equatorial climate characterised by a temperature range of 18–27 °C [25] and two seasons: a short dry season (November–February) and a long rainy season (March–October). *P. falciparum* is the main malaria parasite species accounting for over 90% of reported malaria, and *Anopheles gambiae* is the main vector species. Malaria transmission is perennial with two peaks—the beginning and end of the rainy season [26].

Although the indigenes of this area are of the Bakweri tribe and part of the Bantu ethnic group [27], its fertile volcanic soils have attracted people from other regions of the country, mainly from the Semi-Bantu ethnic group of the North West. Plank houses are common although cement block houses predominate in the village. Subsistence farming and horticulture is the mainstay of the village communities, which rely mainly on agriculture for their livelihood.

The participants included pre- and school age children of both sexes aged 6 months to 14 years old. They weighed > 5 kg and were free from other clinical conditions not related to severe malaria and the sickle cell disease. Exclusion criteria in this study included children with severe malaria (unable to drink or breastfeed, vomiting more than twice in the preceding 24 h before presentation, recent history of convulsions, unconscious state or unable to sit or stand and other diseases requiring hospital admission) [2, 4].

### Study design

The study was a cross-sectional community-based design carried out during the peak malaria transmission period in the Mount Cameroon area of April and May 2018 [26]. Following administrative clearances and ethical approval for the study, informed consent/assent forms and information sheets explaining the purpose, risks and benefits of the study were given to parents/caregivers. Participants were invited to the temporary data collection location in each neighbourhood, and coordination was organised by the head/leader of a block within a neighbourhood. The study team proceeded for sample collection upon obtaining consent/assent from the participants. Ensuing administration of a structured questionnaire, body temperature was measured, and blood sample was collected from each child for malaria parasite identification/quantification and a complete blood count assessment. The sample size was calculated using the 21% prevalence of malaria parasites in this study area [28]. Sample size was determined using the formula *n* = *Z*^2^*pq*/*d*^2^ [29] where *n* = the sample size required, *z* = 1.96: which is the standard normal deviate (for a 95% confidence interval, CI), *p* = 66.2%: proportion of malaria prevalence, *q* = 1–*p*: proportion of malaria negative children and *d* = acceptable error willing to be committed. The minimum sample size was estimated as *n* = 255. The sample size was then increased by 25% to a minimum of 319 participants to account for anticipated non-respondents, incomplete data entry and loss of samples due to blood clotting. A convenience sampling method was used in all the blocks within the study area until the required sample was attained.

### Collection of data

Information on malaria knowledge and preventive methods was obtained from the participants using a pre-tested, structured questionnaire. The questions included demographics (sex, age, literacy level, occupation and marital status of parents/caregivers), malaria knowledge (sign/symptoms, complications, transmission) and prevention methods as well as the history of fever.

The axillary temperature was measured using an electronic thermometer and fever was defined as temperature ≥ 37.5 °C [30].

### Laboratory methods

Three to four millilitres (ml) of venous blood was collected from each child using sterile disposable syringes. Drops of whole blood were dispensed immediately on RDT kits using a sample applicator and on slides for the preparation of thick and thin blood films. The remaining blood was dispensed into labelled ethylenediamine tetra-acetate tubes and placed in a cool box for transportation to the Malaria Research Laboratory, University of Buea, for a full blood count analysis. The air-dried thin blood film was fixed in absolute methanol, and both thick and thin blood films were stained using 10% Giemsa solution for 20 min. Each blood film was independently examined microscopically by two well-trained and well-experienced parasitologist, following standard procedure for the detection and identification of malaria parasites [31]. Slides were considered positive when schizonts, trophozoites and/or gametocytes of *Plasmodium* were observed on the blood film. Parasite density was determined on thick blood film by systematically counting the number of parasites per 200 white blood cells and multiplying the parasite count with the participants’ white blood cell count obtained from the full blood count analysis.

A complete haematological assessment of each blood sample was performed using the MINRAY 2800 BC auto-haematology analyser, following the manufacturer’s instructions, and anaemia was defined as Hb < 11.0 g/dL [32].

### The CareStart™ antigen rapid diagnostic test

The CareStart™ Malaria HRP2 pf (CAT NO: G0141, ACCESSBIO) Ag RDT is a chromatographic test for in vitro diagnosis. It has a long shelf-life of more than 1 year, with storage conditions ranging from 1 to 40 °C. Each kit is composed of 25 chromatographic test strips fixed in a cassette, lysis buffer, a pack of 25 lancets and disposable alcohol swabs saturated with 70% isopropyl for disinfection. The CareStart™ Malaria Pf (HRP2) Ag RDT contains a membrane strip pre-coated with a monoclonal antibody as a single line across the test strip conjugated to a signal, typically colloidal gold. The monoclonal antibody is specific to HRP2 of *P. falciparum.*

Each CareStart™ Malaria Pf (HRP2) Ag RDT device was labelled with an identification code like that used on the slide for each study participant. Approximately 5 μL of venous blood was added onto the test device window using the sample applicator provided with the kit. This was followed by adding 2 drops (60 μL) of reagent buffer. The device was allowed untouched for 20 min at room temperature, and the result was recorded as per the instruction of the manufacturer. Negative results were recorded when only a band appeared on the control area whereas the presence of two bands, one band in the control area and the other band in the test area were recorded as a positive result for *P. falciparum.*

### Definitions and endpoints

- Asymptomatic malaria parasitaemia was defined as the presence of *Plasmodium* with an axillary temperature of < 37.5 °C.
- Clinical malaria parasitaemia was defined as the presence of *Plasmodium*, with an axillary temperature of ≥ 37.5 °C, joint pains, vomiting, headache, diarrhoea, chills.
- True positive (TP): Have the disease and test positive.
- False positive (FP): Do not have the disease but test positive.
- True negative (TN): Do not have the disease and test negative.
- False negative (FN): Have the disease but test negative.
- Sensitivity (Se) of the test is the ability of the test to identify correctly those who have the disease (true positive rate). Se = TP/(TP + FN) [33].
- Specificity (Sp) of the test is the ability of the test to identify correctly those who do not have the disease (true negative rate). Sp = TN/(TN + FP) [33].
- Positive predictive value (PPV) is the probability that a disease is present when the test is positive. PPV = TP/(TP + FP) [33].
- Negative predictive value (NPV) is the probability that the disease is not present when the test is negative. NPV = TN/(TN + FN) [33].
- Accuracy (Acc) is the overall probability that a patient will be correctly classified. Acc = (TP + TN)/(TP + TN + FP + FN).

### Statistical analysis

Data collected was cleaned up and analysed using the IBM-Statistical Package for Social Sciences (IBM-SPSS) version 20 and Epi-info version 7. Continuous variables were summarised into means and standard deviations (SD), and categorical variables reported as frequencies and percentages were used to evaluate the descriptive statistics. The differences in proportions were evaluated using Pearson’s chi-square (*χ*^2^). Group means were compared using analysis of variance (ANOVA), and parasite density was log-transformed before analysis. Using light microscopy of Giemsa-stained thick blood film as the gold standard, the RDT Se, Sp, false positive rate, false negative rate, PPV, NPV and Acc were calculated. Inter-test agreement for both results of positive and negative readings was expressed by the percentage of overall agreement and kappa value (*κ*) for the agreement between malaria RDTs and the reference method (microscopy). The detection limit was calculated from the sample with lowest parasitaemia but highest sensitivity and specificity. Associations between predictor variables and primary outcomes were assessed using both bivariate and multivariate logistic regression analysis. Odds ratios (ORs) and 95% confidence intervals (CIs) were computed. Any covariate with a *P* value < 0.2 in the bivariate analysis was subsequently included in the final multivariable logistic model. Significant levels were measured at 95% CI with the level of significance set at *P* < 0.05.

## Results

### Characteristics of study participants

The baseline characteristics of the study population are shown in Table 1. A total of 491 children aged between 6 months and 14 years participated in the study. There were more females (55.6%) than males (44.6%), and most of the participants were less than 5 years (43.6%) old. A greater proportion of parents/caregivers of the children had a primary level of education (51.8%) followed by those with secondary school education (31.6%). The proportion of children who slept under a long-lasting mosquito net the previous night before the survey was 62.3%. On microscopic examination, *P. falciparum* infection was present in 27.7% of the children, while using RDT, malaria was diagnosed in 39.7% of participants. Fever and anaemia were observed in 3.7% and 72.7% of the children, respectively.

**Table 1:** Baseline characteristics of study population

Parameter	Total
% (*N*)	100 (491)
Sex	
Male	44.4 (218)
Female	55.6 (273)
Age groups (years)	
≤ 4	43.6 (214)
5–8	39.1 (192)
≥ 9	17.3 (85)
Mean age ± SD in years	5.20 ± 3.25
Mean temperature ± SD in °C	36.37 ± 0.77
Fever status	
Pyrexia (≥ 37.5 °C)	3.7 (18)
Normal (< 37.5 °C)	96.3 (473)
Educational level of parent/caregiver	
No formal education	12.0 (39)
Primary	51.8 (169)
Secondary	31.6 (103)
Tertiary	4.6 (15)
Mosquito bed net use	
Yes	62.3 (306)
No	37.7 (185)
Malaria parasite prevalence by microscopy	27.7 (136)
Malaria parasite prevalence by RDT	39.7 (195)
Geometric mean parasite density (range)	187 (70–1162)
Prevalence of anaemia	72.7 (357)

### *P. falciparum* prevalence by microscopy and RDT

The prevalence of falciparum malaria among the 491 children varied with sex as shown in Table 2. By microscopy, malaria parasite prevalence was higher in females (31.9%) than males (22.5%) and the difference was significant at *P* = 0.021, whereas by RDT, the difference in prevalence of malaria parasite between males (35.8%) and females (42.9%) was not statistically significant (*P* = 0.111). Conversely, the geometric mean parasite density (GMPD) was significantly higher (*P* = 0.025) in males (218 parasites/μL of blood) than females (172 parasites/μL of blood). A significant difference in *P. falciparum* infection by microscopy (*P* = 0.002) and RDT (*P* = 0.001) was observed with age, with the ≤ 4 years age group having the highest malaria prevalence (31.9% by microscopy and 47.4% by RDT), followed by the 5–8 years age group (29.9% by microscopy and 38.1% by RDT) and least by the ≥ 9 years age group (11.9% by microscopy and 23.8% by RDT). In addition, children ≤ 4 years of age had the highest GMPD (224/μL of blood), followed by the 5–8 years age group (159/μL of blood) and lastly the 9 years age group (141/μL of blood).

**Table 2:** Influence of sex, age, educational level of parents, mosquito bed net use and anaemic status on the prevalence of *P. falciparum* by microscopy and RDT

Parameter	No. examined	Microscopy	RDT	GMPD (range)/μL of blood
		Prevalence (*n*)	Prevalence (*n*)	
Sex				
Male	218	22.5 (49)	35.8 (78)	218 (70–1162)
female	273	31.9 (87)	42.9 (117)	172 (80–1122)
*P* value		0.021^*^	0.111	0.025^*a^
Age group in years				
≤ 4	213	31.9 (68)	47.4 (101)	224 (82–1162)
5–8	194	29.9 (58)	38.1 (74)	159 (70–1054)
≥ 9	84	11.9 (10)	23.8 (20)	141 (82–363)
*P* value		0.002^**^	0.001^**^	0.003^**b^
Educational level of parent				
No formal	68	63.2 (43)	57.4 (39)	276 (93–1162)
Primary	169	20.7 (35)	45.6 (77)	165 (90–1102)
Secondary	103	29.1 (30)	48.5 (50)	210 (74–1054)
Tertiary	78	9.0 (7)	14.1 (11)	166 (82–366)
*P* value		0.001^**^	0.001^**^	0.173^b^
Mosquito bed net use				
Yes	306	15.4 (47)	35.6 (109)	192 (90–1162)
No	184	48.4 (89)	46.7 (86)	185 (70–1122)
*P* value		0.001^**^	0.015^*^	0.562^a^
Anaemic status				
Anaemic	357	30.3 (108)	44.5 (159)	194 (74–1162)
Non-anaemic	134	20.9 (28)	26.9 (36)	165 (70–1054)
*P* value		0.039^*^	< 0.001^***^	0.226^a^

^*^Significant at *P* < 0.05 level, ^**^significant at *P* < 0.01, ^***^significant at *P* < 0.001. Comparisons of proportions by *χ*^2^ and means were compared by ^a^Mann-Whitney *U* test and ^b^Kruskal-Wallis test after log transformation

In both microscopy and by RDT, the prevalence of *P. falciparum* infection was highest in children whose parents had no formal level of education (63.2% by microscopy and 57.4% by RDT), while children whose parents had tertiary level of education had the least prevalence (9.0% by microscopy and 14.1% by RDT), and the difference was significant at *P* < 0.001 and *P* < 0.001 respectively (Table 2).

Children who used mosquito bed net had a significantly lower *P. falciparum* infection prevalence (15.4% by microscopy and 35.6% by RDT) when compared with children who did not use a bed net (48.4% by microscopy and 46.7% by RDT).

*P. falciparum* infection prevalence was significantly higher by microscopy (*P* = 0.039) and RDT (*P* < 0.001) in children with anaemia (30.3% by microscopy and 44.5% by RDT) than those non-anaemic (20.9% by microscopy and 26.9% by RDT) as shown in Table 2.

### Validity of CareStart™ to diagnose *P. falciparum* and concurrence with microscopy

The CareStart™ Malaria HRP2 pf Ag RDT had a sensitivity of 82.4% (95% CI:74.9–88.4%) and specificity of 76.6% (95% CI 71.9–80.9%). The PPV, NPV and Acc were 57.4% (95% CI 52.4–62.3%), 91.9% (95% CI 88.7–94.2%) and 78.2% (95% CI 74.3–81.8), respectively. False positive results were observed in 42.6% (83) of children with microscopy negative results for *P. falciparum,* while the false negative rate was 8.1% (24) among the children as shown in Table 3. The measure of agreement kappa (*κ*) between microscopy and CareStart™ Malaria HRP2 pf Ag RDT (G0141) was 0.52.

**Table 3:** RDT validity to diagnose *Plasmodium falciparum* malaria

Characteristic	Performance 95% CI
Sensitivity	82.4% (74.9–88.4)
Specificity	76.6% (71.9–80.9)
PPV	57.4% (52.4–62.3)
NPV	91.9% (88.7–94.2)
Accuracy	78.2% (74.3–81.8)

Kappa (*κ*) = 0.52

### Predictors of CareStart™ malaria HRP2 pf ag RDT positivity

There was a statistically significant dependence of the positivity of the RDT on clinical malaria parasitaemia (*P* < 0.001), fever (*P* < 0.004) and parasite density (*P* < 0.001). The positivity of the RDT was 100% for clinical malaria parasitaemia (presence of *Plasmodium*, with an axillary temperature of ≥ 37.5 °C) and 72.2% among febrile children (temperature of ≥ 37.5 °C, regardless of the presence of *Plasmodium*). The RDT demonstrated a sensitivity of 96.1% for parasite densities above 200 parasites/μL of blood (Table 4).

**Table 4:** *P. falciparum* infection presentation, fever status and RDT outcome

Parameter	Number examined	RDT		*P* value
		Positive % (*n*)	Negative % (*n*)	
*P. falciparum* infection				
With fever	12	100.0 (12)	0.0 (0)	< 0.001^***^
Without fever	124	80.6 (100)	19.4 (24)	
Fever status				
Febrile	18	72.2 (13)	27.8 (5)	0.004^**^
Afebrile	473	38.5 (182)	61.5 (291)	
Parasite density/μL of blood				
< 200 parasites	84	73.8 (62)	26.2 (22)	0.001^**^
≥ 200 parasites	51	96.1 (49)	3.9 (2)	

^**^Statistically significant at *P* < 0.01, ^***^ statistically significant at *P* < 0.001

A multivariate analysis demonstrated that children who were febrile (*P* < 0.001), less than 5 years old (*P* < 0.02), those who had fever within a month (*P* < 0.001), and those anaemic were more likely to have a positive RDT result. Febrile children were 6.8 times more likely to have a positive RDT for *P. falciparum* than non-febrile children while those under 5 years of age were 2 times at odds of having a positive RDT than their contemporaries. In addition, children with a history of fever within a month at the time of the survey were 4.57 times more likely to be positive for *P. falciparum* infection by RDT when compared with children without fever. Moreover, those with anaemia were 2 times at higher odds of having a positive outcome with RDT than non-anaemic children as shown in Table 5.

**Table 5:** Logistic regression model examining factors associated with malaria RDT positivity

Variables	*N*	Prevalence by RDT (*n*)	Bivariate logistic regression		Multivariate logistic regression	
			COR (95% CI)	*P* value	AOR	*P* value
Fever status						
Afebrile	473	38.5 (182)	Reference		Reference	
Febrile	18	72.2 (13)	4.16 (1.46–11.85)	0.007^**^	6.67 (2.13–20.86)	< 0.001^***^
Age group (years)						
≥ 9	85	23.5 (20)	Reference		Reference	
5–8	192	38.0 (73)	1.97 (1.11–3.52)	0.02^*^	1.42 (0.76–2.66)	0.28
≤ 4	214	47.7 (102)	2.89 (1.63–5.10)	< 0.001^***^	2.06 (1.11–3.84)	0.02^*^
Gender						
Female	273	42.9 (117)	Reference			
Male	218	35.8 (78)	1.35 (0.93–1.94)	0.11	1.38 (0.92–2.06)	0.12
Fever within a month						
No fever	323	27.9 (90)	Reference			
Fever	168	62.5 (105)	4.31 (2.90–6.41)	< 0.001^***^	4.57 (3.02–6.92)	< 0.001^***^
Anaemia						
Negative	133	27.1 (36)	Reference			
Positive	357	44.5 (159)	2.18 (1.41–3.38)	< 0.001^***^	2.11 (1.29–3.44)	0.002^**^

^*^Statistically significant at *P* < 0.05, ^**^statistically significant at *P* < 0.01, ^***^statistically significant at *P* < 0.001

### Receiver operating characteristic (ROC) curve

The accuracy of malaria parasitaemia as assessed by the area under the ROC curve to predict malaria by RDT was 75.4% (95% CI, 70.6–80.1). The optimal cutoff for the diagnosis of malaria by RDT was 77 parasites/μL of blood.

## Discussion

The determination of the diagnostic performance characteristics of the rapid immune-chromatographic methods is essential given the need for accurate and prompt diagnosis before treatment. With the availability of various RDTs, a limitation of comparative field trials and the heterogeneity of the transmission and epidemiology of malaria in Cameroon, the findings of the study provide essential information for an informed decision to be made by the NMCP.

A prevalence of 27.7% for malaria was observed by microscopy in this study population. This prevalence was similar to the 29.6% reported by Apinjo et al. [28] in children < 15 years of age in the South West Region of Cameroon. On the contrary, higher prevalence of 46.7% and 36.9% in children less than 15 years has been reported in other areas in the Mount Cameroon area [2]. Besides a decline in prevalence, this study also observed a remarkable fall in geometric mean parasite density from 2131 parasites/μL [25] to 187 parasites/μL in this population. Similarly, the proportion of febrile children in this population was 3.4% and has also experienced substantial decrease from 27.1% [25] in Dibanda and 41.9% [34] in different communities of the Mount Cameroon area. The decline in morbidity in the study area is not surprising because recently, Dibanda has been experiencing urban transformation from a farm land site/village setting into a settlement area “new layout.” Various authors have postulated that increased urbanisation reduces the number of breeding places for *Anopheles* mosquitoes and consequently malaria transmission [35, 36]. Also, in Dibanda, there is a general change in house type from predominantly plank houses [28] to cement block houses which may have played a role in curbing the disease burden. It has been reported that a plank house has crevices for mosquito entry [37] and provides a favourable microenvironment for mosquitoes [38]. In addition, the decrease in malaria morbidity could also be the result of sustained control measures as reported by WHO [2] and Sumbele et al. [4].

Malaria prevalence was higher in females than males by microscopy and the findings are in line with Kimbi et al. [32] who reported that females spend more time outdoors at dusk and dawn than males to perform household chores and as such are more exposed to mosquito bites. It is not surprising that children under 5 years had the highest parasitaemia when compared with the older children. Children under 5 years are more vulnerable to the disease in areas of high transmission [2, 30, 34] as naturally acquired immunity builds up in older children following repeated exposure to the parasite. In line with the findings of Ebai et al. [34] in other parts of the Mount Cameroon area, children from individuals with no formal or with primary education were more infected with the malaria parasite than those with secondary or tertiary education. Higher levels of education are generally associated with improved knowledge and practices in relation to appropriate prevention and treatment strategies.

The high prevalence of anaemia (72.7%) in children less than 15 years is not an uncommon finding in this part of the country, and this highlights anaemia as a major public health problem among the population in this area. The relationship between malaria parasitaemia and anaemia is well established in previous studies [2, 30, 39]. However, findings in the area [30] reported a low risk of anaemia attributable to malaria highlighting the insidious and important contribution of other inflammatory infections or diseases which were not investigated in the study.

In the present study, CareStart RDT sensitivity (82.4) and specificity (76.6) did not reach the values indicated by the manufacturer (98% and 97.5%, respectively). The PPV was 57.4%, and the NPV was 91.9%. This could be explained by the difference between populations used to estimate these parameters. In the present study, we evaluated a population with all levels of parasitaemia, whereas the manufacturer’s tests were done on samples with a density of > 200 parasites/μL of blood. The results from this study are consistent with similar studies reported in Africa. A study by Wanja et al. [40] in Western Kenya showed that CareStart (G0181, G0131 and G0141) had a sensitivity range of 86.9–95.48%, specificity range of 71.2–81.4%, PPV range of 59.2–72.8 and NPV range of 91.9–97.9. Also, Maltha et al. [41], in a reference setting showed the sensitivity of the CareStart Malaria HRP2/pan kit for the detection of *P. falciparum* to be 84.8–92.0%. Findings from this study demonstrated a higher CareStart RDT (G0141) performance characteristics when compared with a previous study carried out by Ndamukong-Nyanga [21] in the Mount Cameroon area (sensitivity = 48.5%, specificity = 84%, PPV = 62.3% and NPV = 75.0%) even though their mean parasite density/μL of blood (2333, range 18–16,000) was higher. However, a lower sensitivity in performance could be attributed to a prozone effect which has been reported as a cause of false negative HRP-2 RDT [42].

On the other hand, a study by Maltha et al. [43] in Burkina Faso demonstrated higher sensitivity, specificity, PPV and NPV of 100%, 70.9%, 69.4% and 100%, respectively, for PfHRP2 detection. Likewise, a study by Diallo et al. [42] in Senegal demonstrated that CareStart™ RDT showed high sensitivity (97.3%) and specificity (94.1%) with PPV and NPV of 97.3 and 94.1%, considering polymerase chain reaction as standard. The higher performance of the HRP2 RDT from these studies was probably due to their study design. Unlike our study which was on non-clinical cases and had as limitation a less sensitive gold standard the Giemsa-stained microscopy when compared with the highly sensitive PCR, these studies were all hospital based and the occurrence of fever was an inclusion criterion.

The sensitivity of the RDT in this study increased with an increase in parasite density achieving a sensitivity of 96.1% at parasite density greater than or equal to 200 parasites/μL of blood, as recommended by WHO [17]. This is in line with several studies [40, 41, 44] where the sensitivity increased to > 95% at parasite densities greater than 200 parasites/μL of blood. This finding confirms the inability of RDTs to reliably detect malaria parasites at very low parasite densities. The CareStart RDT was positive for all febrile children with malaria (100%). This study also demonstrated that febrile children were 6.7 times at higher odds to be positive by RDT when compared with afebrile children. A sensitivity of 100% was reported by Maltha et al. [43] among hospitalised febrile children in Burkina Faso. Moreover, studies by Wilson [45] reported that patients with febrile illness in endemic areas are likely to be diagnosed with malaria. While the CareStart RDT could be considered a useful diagnostic tool for ruling out malaria in cases where good Giemsa microscopy is not available or appropriate in the health facility, its applications in communities and population-based screening with a higher proportion of asymptomatic cases are limited.

Findings from the study showed a low positive predictive value (57.4%) indicating a high false positive rate. This could probably be due to prior infection and subsequent effective treatment as reported in the structured questionnaires. This study indicated that children who had a history of fever within a month prior to the survey were 4.6 times more likely to be positive by RDT. The HRP2 antigen detected by this RDT has been demonstrated in several studies to persist in the blood stream between 14 and 30 days before being cleared completely [46, 47]. Moreover, as a limitation in the study, rheumatoid factor which was not measured has been suggested to produce false positive due to binding IgG [48].

The relatively high negative predictive value (91.9%) in this study indicates the likelihood of the RDT to correctly identify a child without malaria as true negative, hence showing a substantive ability to differentiate between children who had malaria and those without. Hence, this RDT could be confidently used to confirm negative test patients as non-malaria patients. The false negative results produced by CareStart (G0141) could be accounted for by deletions or mutations of the HRP2 gene such that the parasite no longer produces the antigen or produces the mutant antigen that is not recognised by antibodies on the test strip [22].

This study revealed that children under 5 years were 2 times more likely to be positive for RDT when compared with older children. Similar results with a decrease in sensitivity of the Paracheck-Pf RDT in older age groups have been reported in Tanzania [49]. This is probably because younger children are more vulnerable to the disease and have higher parasite loads when compared with older children. This observation is in line with reports from other studies [2, 50].

Findings in this study also revealed higher odds of having a positive RDT among anaemic than non-anaemic children. This is probably because of the likelihood of anaemic children having *Plasmodium* infection and a higher parasite load. Malaria has been reported by several authors to contribute substantially to anaemia in malaria-endemic regions [39, 51] and this study attests to this.

## Conclusions

The overall concurrence of CareStart™ Malaria HRP2 pf (G0141) with microscopy in population screening for *P. falciparum* infection in the community is moderate and is most useful at parasitaemia ≥ 200 parasites/μL of blood and presentation with feverin in this malaria-endemic region. The RDT has proven to be very effective as a diagnostic test for confirmation of clinical cases of malaria. However, its applications in population-based screening in the community with a higher proportion of asymptomatic cases are limited. Control measures involving population screening should be complemented with microscopy for non-clinical cases for proper case management. Considering that RDT positivity is significantly associated with fever, age less than 5 years, history of fever within a month and anaemia, its wide use can significantly improve the quality of malaria case management by avoiding indiscriminate use of antimalarial drugs for parasite negative patients in malaria-endemic regions. As a recommendation, the NMCP should ensure more assessment for the performance evaluation of the different malaria RDT brands available in the Cameroon market for good management of the disease.

## Abbreviations

Acc: Accuracy
ACT: Artemisinin-based combination therapy
Ag: Antigen
ANOVA: Analysis of variance
CI: Confidence intervals
EDTA: Ethylenediaminetetraacetate
FIND: Foundation for Innovative Diagnostics
FN: False negative
FP: False positive
GMPD: Geometric mean parasite density
Hb: Haemoglobin
HRP2: Histidine-rich protein 2
NMCP: National Malaria Control Program
NPV: Negative predictive values
ORs: Odds ratios
PfHRP2: *Plasmodium falciparum* histidine-rich protein 2
PPV: Positive predictive values
RDT: Rapid diagnostic tests
ROC: Receiver operating characteristic
SD: Standard deviations
Se: Sensitivity
Sp: Specificity
TN: True negative
TP: True positive
WHO: World Health Organization
*κ*: Kappa value

## References

[1] WHO. World malaria report 2017: World Health Organization; 2017. http://www.who.int/maaria/publications/world-malaria-report-2017/report/en/. Accessed 29 Nov 2018

[2] Teh RN, Sumbele IUN, Meduke DN, Ojong ST, Kimbi HK (2018). Malaria parasitaemia, anaemia and malnutrition in children less than 15 years residing in different altitudes along the slope of Mount Cameroon: prevalence, intensity and risk factors. Malaria J..

[3] Fokam EB, Kindzeka GF, Ngimuh L, Dzi KT, Wanji S (2017). Determination of the predictive factors of long-lasting insecticide-treated net ownership and utilisation in the Bamenda Health District of Cameroon. BMC Public Health..

[4] Sumbele IU, Ning TR, Bopda OS, Nkuo-Akenji T (2014). Variation in malariometric and red cell indices in children in the Mount Cameroon area following enhanced malaria control measures: evidence from a repeated cross-sectional study. Malar J..

[5] Kimbi HK, Nkesa SB, Ndamukong-Nyanga JL, Sumbele IU, Atashili J, Atanga MB (2014). Knowledge and perceptions towards malaria prevention among vulnerable groups in the Buea Health District, Cameroon. BMC Public Health..

[6] Ndo C, Menze-Djantio B, Antonio-Nkondjio C (2011). Awareness, attitudes and prevention of malaria in the cities of Douala and Yaoundé (Cameroon). Parasit Vectors..

[7] Ndong IC, van Reenen M, Boakye DA, Mbacham WF, Grobler AF (2015). Trends in malaria case management following changes in the treatment policy to artemisinin combination therapy at the Mbakong Health Centre, Cameroon 2006–2012: a retrospective study. Acta Trop..

[8] WHO: Malaria rapid diagnostic test performance: results of WHO product testing of malaria RDTs: round 8 (2016–2018); 2018.https://www.who.int/malaria/publications/atoz/9789241507554-summary_eng.pdf. Accessed 20 Jan 2019.

[9] WHO (2014). World Malaria Report.

[10] Kimbi HK, Ajeagah HU, Keka FC, Lum E, Nyabeyeu HN, Tonga CF (2012). Asymptomatic malaria in school children and evaluation of the performance characteristics of the PartecCyscope® in the Mount Cameroon region. J BacteriolParasitol..

[11] Singh N, Mishra AK, Shukla MM, Chand SK, Bharti PK (2005). Diagnostic and prognostic utility of an inexpensive rapid on site malaria diagnostic test (ParaHIT f) among ethnic tribal population in areas of high, low and no transmission in central India. BMC Infect Dis..

[12] Moody A (2002). Rapid diagnostic tests for malaria parasites. ClinMicrobiol Rev..

[13] Ansah EK, Epokor M, Whitty CJ, Yeung S, Hansen KS (2013). Cost-effectiveness analysis of introducing RDTs for malaria diagnosis as compared to microscopy and presumptive diagnosis in central and peripheral public health facilities in Ghana. Am J Trop Med Hyg..

[14] Perkins MD, Bell DR (2008). Working without a blindfold: the critical role of diagnostics in malaria control. MalarJ..

[15] Rafael ME, Taylor T, Magill A, Lim Y-W, Girosi F, Allan R (2006). Reducing the burden of childhood malaria in Africa: the role of improved. Nature..

[16] White NJ (2004). Antimalarial drug resistance. J Clin Invest..

[17] Bell D, Cunningham J: Malaria rapid diagnostic test performance; results of WHO product testing of malaria RDTs: round 3 (2010–2011). 2011.https:apps.who.int/iris/bitstream/handle/10665/204118/9789241510035_eng.pdf;jsessionid=430E08DE8C95D56CDB354E49DA1FD6E2sequence=1. Accessed 07 Dec 2018.

[18] Rosanas-Urgell A, Mueller D, Betuela I, Barnadas C, Iga J, Zimmerman PA (2010). Comparison of diagnostic methods for the detection and quantification of the four sympatric Plasmodium species in field samples from Papua New Guinea. MalarJ..

[19] Visser T, Daily J, Hotte N, Dolkart C, Cunningham J, Yadav P (2015). Rapid diagnostic tests for malaria. Bull World Health Organ..

[20] WHO (2017). Malaria rapid diagnostic test performance: summary results of WHO producttesting of malaria RDTs: round 1–7 (2008–2016).

[21] Ndamukong-Nyanga JL, Kimbi HK, Sumbele IUN, Emmaculate L, Nweboh MN, Nana Y (2014). Assessing the performance characteristics of the “CareStartTM Malaria HRP2 Pf (CAT NO: G0141, ACCESSBIO)” rapid diagnostic test for asymptomatic malaria in mutengene, Cameroon. Int J Trop Dis Health..

[22] Kumar N, Pande V, Bhatt RM, Shah NK, Mishra N, Srivastava B (2013). Genetic deletion of HRP2 and HRP3 in Indian *Plasmodium falciparum* population and false negative malaria rapid diagnostic test. Acta Trop..

[23] Wanji S, Kimbi HK, Eyong JE, Tendongfor N, Ndamukong JL (2008). Performance and usefulness of the Hexagon rapid diagnostic test in children with asymptomatic malaria living in the Mount Cameroon Region. Malar J..

[24] Mangham LJ, Cundill B, Achonduh OA, Ambebila JN, Lele AK, Metoh TN (2012). Malaria prevalence and treatment of febrile patients at health facilities and medicine retailers in Cameroon. Trop Med Int Health..

[25] Ndamukong-Nyanga JL, Kimbi HK, Sumbele IUN, Nana Y, Bertek SC, Ndamukong KJ (2015). A cross-sectional study on the influence of altitude and urbanisation on co-infection of malaria and soil-transmitted helminths in Fako Division, South West Cameroon. Int J Trop Dis Health..

[26] Wanji S, Kengne-Ouafo AJ, Eyong EEJ, Kimbi HK, Tendongfor N, Ndamukong-Nyanga JL (2012). Genetic diversity of *Plasmodium falciparum*merozoite surface protein-1 block 2 in sites of contrasting altitudes and malaria endemicities in the Mount Cameroon Region. Am J Trop Med Hyg..

[27] Ngwa G (2000). Geography for the Republic of Cameroon.

[28] Apinjoh TO, Anchang-Kimbi JK, Mugri RN, Tangoh DA, Nyingchu RV, Chi HF (2015). The effect of insecticide treated nets (ITNs) on *Plasmodium falciparum*infection in rural and semiurban communities in the South West Region of Cameroon. PLoS ONE..

[29] Manly BF. The design and analysis of research studies. Cambridge: Cambridge University Press; 1992.

[30] Sumbele IUN, Sama SO, Kimbi HK, Taiwe GS (2016). Malaria, moderate to severe anaemia, and malarial anaemia in children at presentation to hospital in the Mount Cameroon area: a cross-sectional study. Anemia..

[31] Cheesbrough M. District laboratory practice in tropical countries. Cambridge: Cambridge university press; 2006.

[32] Kimbi HK, Sumbele IUN, Nweboh M, Anchang-Kimbi JK, Lum E, Nana Y (2013). Malaria and haematologic parameters of pupils at different altitudes along the slope of Mount Cameroon: a cross-sectional study. Malar J..

[33] Gordis L (2014). Epidemiology.

[34] Ebai CB, Kimbi HK, Sumbele IUN, Yunga JE, Lehman LG (2016). Epidemiology of *Plasmodium falciparum* malaria in the Ikata-Likoko area of Mount Cameroon: a cross sectional study. Int J Trop Dis Health..

[35] Kimbi HK, Nana Y, Sumbele IN, Anchang-Kimbi JK, Lum E, Tonga C (2013). Environmental factors and preventive methods against malaria parasite prevalence in rural Bomaka and urban Molyko, Southwest Cameroon. J Bacteriol Parasitol.

[36] Keating J, Macintyre K, Mbogo C, Githeko A, Regens JL, Swalm C (2003). A geographic sampling strategy for studying relationships between human activity and malaria vectors in urban Africa. Am J Trop Med Hyg..

[37] Kirby MJ, Green C, Milligan PM, Sismanidis C, Jasseh M, Conway DJ (2008). Risk factors for house-entry by malaria vectors in a rural town and satellite villages in The Gambia. Malar J..

[38] Ghebreyesus TA, Haile M, Witten KH, Getachew A, Yohannes M, Lindsay SW (2000). Household risk factors for malaria among children in the Ethiopian highlands. Trans R Soc Trop Med Hyg..

[39] Achidi EA, Anchang-Kimbi JK, Mugri RN, Ngwai AN, Yafi CN, Apinjoh TO (2012). Severe and uncomplicated falciparum malaria in children from three regions and three ethnic groups in Cameroon: prospective study. Malar J.

[40] Wanja EW, Kuya N, Moranga C, Hickman M, Johnson JD, Moseti C (2016). Field evaluation of diagnostic performance of malaria rapid diagnostic tests in western Kenya. Malar J.

[41] Maltha J, Gillet P, Bottieau E, Cnops L, van Esbroeck M, Jacobs J (2010). Evaluation of a rapid diagnostic test (CareStart™ Malaria HRP-2/pLDH (Pf/pan) Combo Test) for the diagnosis of malaria in a reference setting. Malar J..

[42] Diongue K, Ndiaye M, Gaye A, Deme A, Badiane AS, Ndiaye D (2017). Evaluation of CareStart™ Malaria HRP2/pLDH (Pf/pan) Combo Test in a malaria low transmission region of Senegal. Malar J..

[43] Maltha J, Guiraud I, Lompo P, Kaboré B, Gillet P, Van Geet C (2014). Accuracy of Pf HRP2 versus Pf-pLDH antigen detection by malaria rapid diagnostic tests in hospitalized children in a seasonal hyperendemic malaria transmission area in Burkina Faso. Malar J..

[44] Kashosi TM, Mutuga JM, Byadunia DS, Mutendela JK, Mulenda B, Mubagwa K (2017). Performance of SD Bioline malaria Ag Pf/pan rapid test in the diagnosis of malaria in South-Kivu, DR Congo. Pan Afr Med J..

[45] Wilson ML (2013). Laboratory diagnosis of malaria: conventional and rapid diagnostic methods. Arch Pathol Lab Med..

[46] Abeku TA, Kristan M, Jones C, Beard J, Mueller DH, Okia M (2008). Determinants of the accuracy of rapid diagnostic tests in malaria case management: evidence from low and moderate transmission settings in the East African highlands. Malar J..

[47] Nyunt MH, Kyaw MP, Win KK, Myint KM, Nyunt KM (2013). Field evaluation of HRP2 and pan pLDH-based immunochromatographic assay in therapeutic monitoring of uncomplicated falciparum malaria in Myanmar. Malar J..

[48] Stauffer WM, Cartwright CP, Olson D, Juni BA, Taylor CM, Bowers SH (2009). Superior diagnostic performance of malaria rapid diagnostic tests as compared to blood smears in US clinical practice. Clin Infect Dis..

[49] Laurent A, Schellenberg J, Shirima K, Ketende SC, Alonso PL, Mshinda H (2010). Performance of HRP-2 based rapid diagnostic test for malaria and its variation with age in an area of intense malaria transmission in southern Tanzania. Malar J..

[50] Sumbele IUN, Bopda OSM, Kimbi HK, Ning TR, Nkuo-Akenji T (2015). Nutritional status of children in a malaria meso endemic area: cross sectional study on prevalence, intensity, predictors, influence on malaria parasitaemia and anaemia severity. BMC Public Health.

[51] Sumbele IUN, Kimbi HK, Ndamukong-Nyanga JL, Nweboh M, Anchang-Kimbi JK, Lum E (2015). Malarial anaemia and anaemia severity in apparently healthy primary school children in urban and rural settings in the Mount Cameroon area: cross sectional survey. PloS One..

